# “Sehat Sahulat Program”: A Leap into the Universal Health Coverage in Pakistan

**DOI:** 10.3390/ijerph19126998

**Published:** 2022-06-07

**Authors:** Syed Shahzad Hasan, Zia Ul Mustafa, Chia Siang Kow, Hamid A. Merchant

**Affiliations:** 1 Department of Pharmacy, University of Huddersfield, Queensgate, Huddersfield HD1 3DH, UK; s.hasan@hud.ac.uk; 2 Department of Pharmacy Services, District Head Quarter Hospital, Pakpattan 57400, Pakistan; zia.ucp@gmail.com; 3 School of Pharmacy, International Medical University, Bukit Jalil, Kuala Lumpur 57000, Malaysia; chiasiang_93@hotmail.com; 4 Health Services Academy, Chak Shahzad, Islamabad 44000, Pakistan

**Keywords:** Universal Health Coverage, World Health Organization, Benazir Income Support Program (BISP), Sehat Insaf Cards, low and middle-income countries

## Abstract

Universal Health Coverage (UHC), initiative from the World Health Organization (WHO), is a means to provide the human right to health by providing essential health services to everyone, enabling disease prevention, treatment, rehabilitation, and palliative care. In line with the WHO recommendations, the UHC was first introduced in Pakistan in Khyber Pakhtunkhwa (KP) province under the name ‘Sehat Sahulat Programme’ (SSP), literally ‘Health Facility Program’ in 2015. The provincial Government in Punjab approved a similar initiative in Punjab, the largest province (by population) of the country, and the program was later rolled out in Islamabad Capital Territory (ICT), Azad and Jammu Kashmir (AJK), Gilgit Baltistan (GB), Sindh, and Baluchistan provinces leaping into the nation-wide coverage. This article provides a current overview of the UHC initiative in Pakistan, analyses its progress in appraising key milestones, and makes recommendations to achieve a robust universal health coverage across Pakistan.

## 1. Introduction

Nearly half of the world’s population is unable to access basic health facilities due to the enormous cost of health services. More than 930 million people spend more than 10% of their household income to access health services. More than 100 million people are pushed into extreme poverty every year due to expenditure in acquiring necessary healthcare [1]. In order to make health services accessible to anyone who needs them anytime, there must be a systematic approach, a skilled healthcare workforce, and a robust policy that can advocate effectively.

In the last decade, there has been much emphasis on the institutions, organizations, physical, financial, and human sources integrated with health services that could meet the health care need of the population. It is estimated that 95% of maternal and child deaths occur in 75 low and middle-income countries (LMICs) that have only 62% of the trained health care workers [2]. Epidemiology of many low and middle-income countries confirmed that the magnitude of diseases is increasing. For example, many communicable diseases emerged in the African region in the last two decades, further stressing their health care system [3]. Non-communicable diseases also carry a huge economic burden in these nations. According to the WHO estimates, non-communicable diseases such as diabetes mellitus, obesity, cancer, and chronic obstructive pulmonary diseases (COPD) are responsible for 80% of the burden of the disease and 70% of the global burden of deaths. At the same time, LMICs are a major contributor to the global diseases and deaths [4]. A sedentary lifestyle, over-urbanization, and unhealthy eating habits (emerging junk food culture) further increased the burden of non-communicable diseases in LMICs [5]. Moreover, the economy in LMICs is under hyperinflation due to lack of continuation of public health policies, political immaturity, inefficient democratic practices, and ineffective health insurance infrastructure that have all led to poor governance, the current sociodemographic situation in LMICs, and inequitable health coverage [6,7].

## 2. The Universal Health Coverage

Universal Health Coverage (UHC), initiative from the World Health Organization (WHO), is a means to provide the human right to health by providing essential health services to everyone, enabling disease prevention, treatment, rehabilitation, and palliative care. The delivery of the services mentioned above require competent and skillful health care workers and a supportive workplace to provide necessary health services effectively. In UHC, every citizen can access necessary healthcare to treat diseases and are protected from the out-of-pocket health expenditures. UHC has been the target of the nations under the umbrella of sustainable development goals (SDGs) since 2015. In 2019, the participating countries ensured the implementation of UHC in the United Nations’ high-level meeting on this subject. The delivery of essential health services to more than one billion people has also been one of the WHO strategic goals for 2022. Under this program, one billion people worldwide needed access to standardized health services every five years from 2015 to 2030. The UHC from WHO will be fully aligned with SDG indicator 3.8.1 (Coverage of essential health services), calculated based on tracer interventions for reproductive, maternal, newborn, and child health; infectious diseases; and noncommunicable diseases, for which the data were already available. Their work on UHC focuses on achieving UHC, including financial risk protection, access to essential healthcare services of acceptable quality, and access to safe, effective, and affordable essential medicines and vaccines for all.

With the active participation of the World Bank, WHO has developed an effective model to track the progress of UHC considering (a) the proportion of the population that can access healthcare services, and (b) the proportion of the population that spends a considerable amount of household income on healthcare [8].

WHO has, therefore, divided UHC into sixteen essential healthcare services under four major indicators. The first indicator is reproductive and newborn health. In this indicator, the reproductive health of the population was provided, including family planning. Antenatal and delivery care was also included in this domain. Moreover, after childbirth, immunization care and subsequent complications such as prevention and treatment from pneumonia care were also included. The second indicator was infectious diseases treatment. In this indicator, infectious diseases such as tuberculosis, human immunodeficiency virus (HIV), prevention techniques against malaria, and sanitation techniques were stressed. The third indicator was non-communicable diseases. Hypertension, diabetes mellitus, cervical cancer, and smoking-related healthcare services are included in this indicator. In addition, health screening, disease prevention, and treatment facilities were also included. Finally, the last indicator was the health services capacity and access. This indicator stressed the availability of basic-level hospitals, appropriate healthcare worker population, access to essential medicines, and health security according to the international norms and regulations [9].

Reich et al. previously classified four distinct groups of LMICs at different points along the UHC ladder; the first group is comprised of countries (e.g., Bangladesh, Ethiopia) which are struggling to integrate the UHC agenda within their national policy, and thus are found at the bottom of the UHC ladder; the second group is comprised of countries (e.g., Ghana, Peru, and Vietnam) with noticeable progress toward UHC that are still experiencing huge gaps in the coverage; the third group is comprised of countries (e.g., Brazil, Thailand, and Turkey), who have attained the key UHC goals but are struggling to sustain [10]. Finally, the fourth group is comprised of countries who achieved UHC in full and need to implement major policy adjustments to address demographic and epidemiological challenges of ageing populations, increasing prevalence of degenerative diseases, and innovations in technology.

This article provides a current overview of UHC initiative in Pakistan, analyses its current progress in appraising key milestones, and makes recommendations to achieve robust universal health coverage across Pakistan.

## 3. Universal Health Coverage in Pakistan

### 3.1. Healthcare in Pakistan

**Figure 1:**
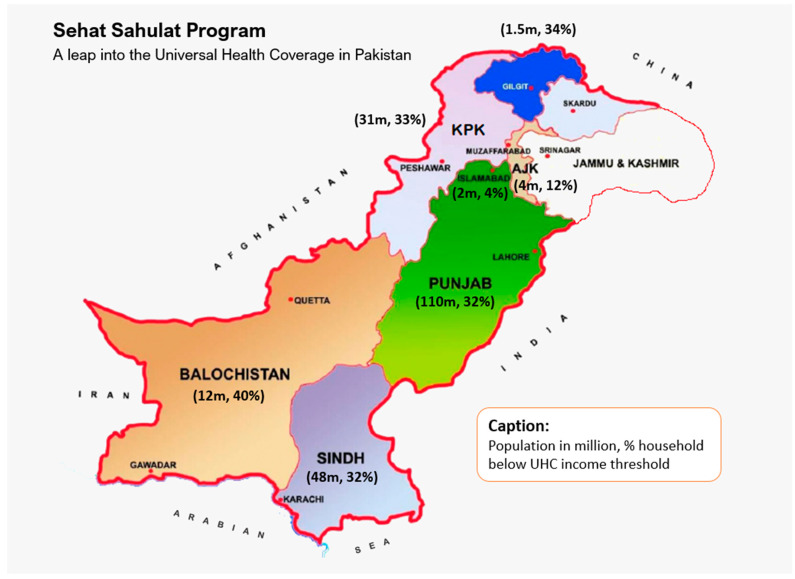
Geographical map of Pakistan showing four major provinces (Sindh, Baluchistan, Khyber Pakhtunkhwa, Punjab), Islamabad Capital territory, and autonomous territories under Pakistan administration (Gilgit Baltistan and Azad and Jammu Kashmir) with population in millions (2017 Census) and percentage of population below UHC income threshold (NSER—Pakistan’s national socioeconomic registry) in each province/territory.

Pakistan, a low to middle-income country (LMIC) in the South Asian region, has a population of more than 220 million [8]. Pakistan is located from 23°35′ to 37°05′ north latitude and 60°50′ to 77°50′ east longitude, touching the Hindukush Mountains in the north and extending from the Pamirs to the Arabian Sea. Pakistan covers 796,095 sq.km and is divided into four major provinces: Punjab, Sindh, Khyber Pakhtunkhwa (KP), and Baluchistan. It also includes Islamabad Capital Territory (ICT), Gilgit Baltistan (GB) and Azad and Jammu Kashmir (AJK), where GB and AJK are autonomous territories administered by Pakistan [11] (Figure 1).

Historically, Pakistan has poor health outcomes amid poor quality of public health facilities, limited access and utilization of health services, poor quality of care, and limited accountability in the public sector. Health indicators such as neonatal mortality rate, infant mortality rate and maternal mortality ratios were among the worst in most rural districts of Pakistan, which constitutes the majority of the population in Pakistan. Only a fraction of the women has access to postnatal care within health center for at least 12 h following birth, with a significant number of births still taking place outside health facilities [12,13]. The health indicators in urban districts are appreciably better due to higher literacy rates and better health awareness among urbanized population, but are mainly driven by the provision of private healthcare providing three-quarters of services that constitute significant out of pocket expenditure to the public at the point of care that is beyond the reach of the majority of Pakistani population. Previously, national health services were under the purview of the federal government that was devolved to provincial authorities following 18th constitutional amendments over a decade ago as part of the sector reforms in hopes to achieve sustainable development goals [14].

### 3.2. Overview of UHC in Pakistan

In line with the WHO recommendations on UHC, the provincial government of the Khyber Pakhtunkhwa (KP) first started the ‘Sehat Sahulat Program’ (SSP), literally ‘Health Facility Program’, under the umbrella of Universal Health Coverage (UHC) in 2015. Under this initiative, the ‘Sehat Insaf Cards’ were issued to the family in three phases to extend the coverage throughout the KP province. The objective of the SSP was to provide healthcare to more than 4 billion residents in all 35 districts of the KP province (Table 1). Under this program, resident can avail the treatment of up to Rs. 1 million (approx. $6000) per family per year in more than 400 public and private sectors of the KP representing about 25% of the health facilities in the province [15]. KP is in the northwestern region of Pakistan and is the smallest province geographically, but it has a population of over 30 million. The province currently faces considerable challenges and political instability confounded by the influx of refugees, violence, and a prolonged state of insecurity that has restricted its economic and social progress. The manufacturing sector is underdeveloped, and forestry and agriculture are the main source of economic activity. As a result, KP produces only 8% of Pakistan’s GDP with a per capita income of $800, i.e., half the national average [16].

The State Life Insurance Corporation (SLIC) administers the SSP program in Pakistan. It is fully subsidized by the government, which pays a fixed premium per eligible family to the SLIC, who then manages the in-patient healthcare expenditure of the registered users. Under the arrangements, ninety per cent of any unspent net premium is refunded to the government at the end of the three-year contract [17].

The actuarial analysis of the SSP was commissioned by the Deutsche Gesellschaft fur Internationale Zusammenarbeit (GIZ) on behalf of the Government of Germany [17]. The analysis looked at the ‘base table’, the basis for the projection model. This reviewed the incidence rates and average costs under different clinical categories such as medical, surgical, or maternity. The cashless scheme for the beneficiary was based on the hospital reimbursement model; there were no exclusions, and all pre-existing conditions were covered for the in-patients. The primary care was restricted to cardiovascular, diabetes or its complications, burns, road accidents, renal disease or dialysis, chronic diseases, organ failure management and oncology, maternity care (capped to Rs. 17,000 per annum per family plus Rs. 350 transportation allowance per discharge for women after discharge from local district hospitals).

They also studied the impact of demographics (age, sex, domicile) on the incidence rates (hospital admissions), cost of claims and the length of hospital stay. It was noted that the incident rates were surprising compared to both the developed (OECD/EU) and new economies (Thailand and Mexico), hence were the key determinant of the claim costs and expenses. The project premiums were based on target populations and UN census data; therefore, the significant changes in the age, sex, or geographical locations can have enormous implications for the claim costs and premiums.

The indicative premium was projected as Rs. 1755 per family per annum for the three years from 2019 to 2021 (equivalent to ~10 US dollars). Using the stress testing model, a −5 to +46% variation in premium was considered up to a maximum of 146% if the utilization were to increase when the scheme matures. This led to a range of Rs. 1674 to 2569 ($9 to $14) per family per annum, discounting the extreme scenario. The report acknowledged that the scheme is likely to go into rapid expansion and the ‘risk premium’ will be insufficient to cover the expected claims and would require an increase for the 2019–2021 period. The pooled financial resources and risks in a unified program are likely to reduce the statistical variability. The report recommended regular monitoring of incidence rates and key variables such as family size and detailed reanalysis biennially. The current model was mainly based on fee-for-service reimbursement of hospital bills, where the report emphasized introducing alternative payment mechanisms to identify inconsistencies and potential fraud. The actuarial analysis was primarily aimed at identifying financial risks by modeling and assumptions that were critical to managing the financial risks. It was, therefore, aimed to maintain the financial sustainability of the program to ensure that upon expansion, an increasing number of citizens will have access to affordable and quality healthcare in Pakistan.

### 3.3. Current Progress

The success of UHC in its preliminary phase has resulted in its expansion to the country’s largest province (by population), with the Government of Punjab allocating Rs. 65 billion to implement SSP on 9 December 2020, with plans to provide a ‘Sehat Insaf Card’ to families in all 36 districts across Punjab [18]. Punjab’s economy is mainly agricultural, but the industry (cottage, textile, ginning, engineering, minerals) makes a substantial contribution. The population density of Punjab is twice the national average; it contains several major cities of Pakistan such as Lahore, Faisalabad, Rawalpindi, Multan, and Gujranwala.

Furthermore, on 28 December 2020, the Prime Minister (PM) of Pakistan announced the extension of the SSP program to the people of Azad Jammu and Kashmir (AJK) in more than 350 eligible hospitals in Pakistan; over a million families were expected to benefit from this initiative [19].

Currently, the SSP only provides coverage to the families living below the poverty line, i.e., earning less than $2/day, equivalent to 32.5 poverty means test scores from the National Socio-economic Registry (NSER) of Pakistan [20] that was originally developed in 2011 to provide a framework for the Benazir Income Support Program (BISP) aimed to offer a social safety net for the poor by cushioning the impact of food, fuel, and financial crises [21]. The eligible residents of Khyber Pakhtunkhwa (KP), Punjab, Azad Jammu and Kashmir (AJK), Gilgit Baltistan (GB), Islamabad Capital Territory (ICT), and some districts across Sindh and Baluchistan benefitted under this arrangement. 

The persons with disabilities and transgender people on the register of the National Database and Registration Authority (NADRA) across Pakistan were offered universal access to the SSP. The program covered a wide range of secondary and tertiary care in hospitals [22]. The program is now rolled out to give universal access to all families domiciled in KP, AJK and Tharparkar district (Sindh) irrespective of their financial status. Families in KP and Tharparkar (Sindh) can also access the health facility through their National Identity Cards without needing to enroll into the SSP system (Table 1).

As of 8 March 2022, over 27 million families are registered with the program across different provinces in Pakistan (Table 1). In addition, a total of over 3.2 million hospital visits have been recorded by the registered beneficiaries [23]. However, the eligibility income thresholds are currently set at approximately $60 per month (about Rs. 9000 per month), which only included families living in extreme poverty, meaning that a major fraction of the population living in poverty could not therefore benefit from the program. Therefore, a new ‘Khushali Survey’ (literally, ‘happiness survey’) was commissioned to redefine the poverty status for eligibility into the program [24].

The rollout in the province of Sindh was lately started, with SSP was offered initially in eight districts, mainly across rural Sindh, benefitting over half a million families. However, the full provincial rollout was still awaited. Sindh is the second-largest economy in Pakistan after Punjab. Karachi, the most populous city and the main financial hub of Pakistan is the provincial capital of the Sindh. It is a significant part of Pakistan’s industrial sector and contains two of its major commercial seaports. Most of the interior Sindh is an agriculture-based economy.

Baluchistan, situated in the southwest of Pakistan, is the largest province of Pakistan geographically but is the least populated. It is a provincial capital, and the largest city is Quetta. The provincial economy is dominated by natural resources, mainly natural gas. Baluchistan is also home to the Gwadar port on the Arabian sea, which has major economic importance. The Baluchistan province is also lagging, with the program being rolled out across six districts with over a hundred thousand families initially registered.

The provincial governments in Sindh and Baluchistan subsequently withdrawn their funding but hope to rejoin the national drive soon to enable their residents to benefit from the universal health coverage enabling Pakistan to meet the WHO milestones by 2023 [24]. In addition, many low and middle-income families in Pakistan do not have the means to fund emergency hospital care. Therefore, the extension of the essential health coverage from those below the poverty line to the low and middle-income families in Pakistan will be a key milestone in establishing a true Universal Health Coverage over the years.

The program has expanded across Punjab province at a very fast pace, particularly extending the coverage to the southern region that has been underprivileged for a long period, with recent extensions to Bahawalpur, Faisalabad, and Multan divisions and further extensions planned in Gujranwala and Sargodha by the end of March 2022, offering coverage to 30 million families in Punjab only [25].

### 3.4. Current Challenges and Recommendations

Following constitutional reforms in 2010, health services was devolved into provincial administration with an aim to provide equitable health access to all citizens of Pakistan, and fairness in health financing and responsiveness to healthcare needs at a local level [14]. The federal government retained its constitutional responsibility of health information, inter-provincial coordination, global health, and health regulation; however, responsibilities to provide and administer the healthcare were devolved into provincial mandate. This posed a critical challenge to the implementation of the UHC that needed an unequivocal commitment from all provincial governments.

Unfortunately, Sindh and Baluchistan governments did not fund the UHC initiatives provincially, a major hindrance in offering UHC to ~60 million citizens resident in Sindh and Baluchistan constituting ~30% of Pakistani population. It is, however, hoped that the provincial governments will start their own provincial initiatives [26], leading to universal health coverage for their residents in the years to come that will help meet national sustainable development goals. The few families across Sindh and Baluchistan, however, have universal access to health under federal initiatives to fund health services to transgender and disabled populations nationally. Tharparkar district in Sindh was another exception that was universally funded under federal initiative to support Tharparkar residents living under extreme poverty in a mostly deserted land. Tharparkar has the lowest human development index in Sindh with the highest population below the poverty line, well below UHC income thresholds. The SSP program was, however, successfully rolled out to full universal access to all families domiciled in KP, AJK and Tharparkar district (Sindh), irrespective of their financial status. The families in KP and Tharparkar can access the health facility through their National Identity Cards without needing to enroll in the SSP system.

One of the key limitations of the program repeatedly complaint about by the families is the incompatibility of the costs of treatment in private sector hospitals and caps within the system; many patients were turned away without any treatment when the family was unable to pay the difference. There had been reports of families travelling from remote districts to provincial capitals for specialized treatments such as chemotherapy and have been denied due to incompatibility between the treatment costs and financial limits set in the system [27]. This would require (a) pulling the public sector health services together in providing similar care at much-reduced costs, and (b) negotiating a reduced price from the private sector as the SSP has the potential to be a major purchaser of the healthcare on behalf of the public.

As the program expands further into urban districts, the risks mitigation strategies recommended by the actuarial analysis are necessary. The higher incidence rates and claims are likely to outpace premiums creating a financial deficit for the state insurer, which would need subsidiaries from other insurances or governmental fundings that may put the whole program at risk.

It was also suggested that extending the program to include private insurers could be beneficial in mitigating the risk in addition to increasing the capacity. The inclusion of private insurers can also help control tiered pricing structure from private hospitals and care providers, where state insurer is at risk of paying more than the over-the-counter price for a private patient. However, the rapid expansion without increasing the capacity and institutional negotiated pricing structure, the potential financial deficit may threaten the long-term sustainability of the program [28].

## 4. Conclusions

UHC, initially introduced in Pakistan in Khyber Pakhtunkhwa (KP) province, has rapidly spread nationally, currently providing universal access to healthcare to all families currently domiciled in KP, AJK and Tharparkar district (Sindh), transgenders (nationally) and peoples with disability (nationally), irrespective of their financial status. The facility is also available to eligible families domiciled in GB and ICT regions, with plans to expand the coverage in GB to universal access in the next phase. The families in Sindh and Baluchistan at large are unable to benefit from the program; however, the Sindh government is already considering to launch their own provincial initiative. The key limitations and challenges that the program currently faced mainly include the incompatibility of the costs of treatments and reimbursement caps and the restricted availability of health facilities and treatment options in rural districts. As the program expands rapidly, the risk mitigation strategies are necessary to avoid a financial deficit amid higher incidence rates and claims outpacing premiums. These strategies may include subsidiaries from other insurances or governmental fundings, inclusion of private insurers, tiered pricing structure and negotiated reimbursement rates with private sector at or below the over-the-counter price. It is feared that a rapid expansion without an adequate increase in the capacity and institutionally negotiated pricing structure could lead to a potential financial deficit, which may threaten the long-term sustainability of the program. Nonetheless, UHC in Pakistan has already shown to increase access to quality healthcare for the most vulnerable in the society and to reduce health disparities. The sustainability of these achievements will require the commitment and continual efforts of all key stakeholders in the government and the private sector.

## Figures and Tables

**Table 1:** Pakistan provincial/territorial demographics and SSP enrollment statistics.

Province/Territories	Population in Million ^a^	Household Below SSP Income Threshold, % ^b^	Number of Families Enrolled, Thousands ^c^	
			6 Mar. 2021	31 Dec. 2021
Baluchistan	12	40	118	118 ^1^
GB	1.5	34	71	90
KP	31	33	817	9300 ^2^
Sindh	48	32	545	557 ^2,3^
Punjab	110	32	5318	5318
AJK	4	12	639	867 ^2^
ICT	2	4	62	62

^a^ 2017 census data, Pakistan Bureau of Statistics; ^b^ from Pakistan’s national socioeconomic registry (NSER); ^c^ Sehat Sahulat Program, Ministry of National Health Services, Regulation and Coordination (NHSR&C); ^1^ provincial Government stopped funding the initiative, currently only 16 registered transgender families have access through federal government initiative; ^2^ the program is now rolled out to full universal access to all families domiciled in KP, AJK and Tharparkar district (Sindh) irrespective of financial status, where families in KP and Tharparkar can access the health facility through their National Identity Cards without needing enrollment into the SSP system; ^3^ provincial government stopped funding the initiative; currently only families in Tharparkar district and transgender families (throughout Sindh) have universal access to SSP through federal government initiative.

## Data Availability

Publicly published data and upto date information on UHC in Pakistan can be found at the Sehat Sahulat Program website (https://www.pmhealthprogram.gov.pk/) and the Punjab Health Initiative Management Company (https://phimc.punjab.gov.pk/).
